# Compressive optic neuropathy (CON) in Graves’ disease caused by hypertrophy of levator and superior rectus muscles: A case report

**DOI:** 10.1097/MD.0000000000025062

**Published:** 2021-04-09

**Authors:** Takahisa Hirokawa, Masashi Mimura, Masahiro Tonari, Yohei Sato, Yasushi Fujita, Junko Matsuo, Hidehiro Oku, Jun Sugasawa, Tsunehiko Ikeda

**Affiliations:** Department of Ophthalmology, Osaka Medical College, Osaka, Japan.

**Keywords:** case report, compressive optic neuropathy, goiter, Graves’ ophthalmopathy, triiodothyronine, upper eyelid retraction

## Abstract

**Rationale:**

Enlargemento of the medial rectus is the most predominant factor of compressive optic neuropathy (CON) in Graves‘ disease. This case report indicates that CON could develop only from the hypertrophic superior levator and superior rectus (SL/SR) muscle in a patient with poorly controlled Graves‘ disease, and described the possible risk of FT_3_-thyrotoxicosis with a prominent goiter to develop the current rare case with a review of the literature.

**Patient concerns:**

A 66-year-old woman undergoing endocrine management of hyperthyroidism with prominent goiter visited the Department of Ophthalmology due to right-eye upper-eyelid retraction.

**Diagnoses:**

At initial presentation, the right and left margin reflex distance-1 (MRD-1) was 3.2 mm and 2.1 mm, respectively, and no proptosis or visual dysfunction was observed. Despite insufficient hormonal regulation, she refused to undergo goiter removal. The upper eyelid retraction gradually worsened to 7.7 mm of MRD-1, followed by the onset of 20 prism diopters (PD) of the right hypertropia, resulting in right-eye CON after 6 months. Her free thyroxin level was 3.88 ng/dl and free triiodothyronine was 24.90 pg/ml. Computed tomography and magnetic resonance imaging showed only SL/SR enlargement in the right orbit.

**Interventions:**

Intravenous steroid and radiation therapy resulted in visual improvement; however, a prominent upper eyelid retraction and 35PD of hypertropia remained in her right eye. Orbital decompression, upper retraction repair, and superior rectus recession were performed to prevent the recurrence of CON and correct any disfigurement.

**Outcomes:**

The combination of conventional intravenous steroid pulse therapy, radiotherapy, and orbital decompression was effective, and no recurrence was observed for more than 1.5-years postoperatively.

**Lessons:**

Enlargement of the SL/SR muscle complex may independently induce the CON. We believe that strict attention should be paid to patients with triiodothyronine thyrotoxicosis with progressive eyelid retraction and hypertropia.

## 1 Introduction

Compressive optic neuropathy (CON) is a rare vision threatening condition caused by compression of the optic nerve at the orbital apex by indurated muscles that reportedly occurs in 5% to 6% of patients with severe thyroid-associated ophthalmopathy,^[1]^ and the severity of thyroid eye disease can correlated with free triiodothyronine (FT_3_), thyroid volume, and cigarette-years.^[2]^ A volumetric study investigating the causative factors of CON reported that enlargement of the medial rectus is the most predominant muscle in the factor, followed by the lateral rectus, and then the complex of the superior levator and superior rectus (SL/SR) muscle.^[3]^

Upper-eyelid retraction, known to occur in approximately 90% of patients afflicted with thyroid ophthalmopathy, is reportedly caused by fibrosis of the levator muscles and by stimulation of the Muller muscle by thyroid hormones, FT_3_, and blood catecholamines or hypertrophy.^[4–6]^

Herein, we report the first report of CON that developed only from hypertrophic SL/SR muscles in a patient with poorly controlled Graves‘ disease, and describe the possible risk of FT_3_-thyrotoxicosis with a prominent goiter to develop the current rare case with a review of the literature.

## 2 Ethic

The study adhered to the principles of the Declaration of Helsinki, and written consent for the report and photographs were obtained from the patient.

## 3 Case report

A 66-year-old woman who had been suffering from hyperthyroidism and was undergoing medical treatment to prescribe methimazole (10–20 mg/day) and potassium iodide (50 mg/day) for 23 years presented at our ophthalmology department after becoming aware of the development of upper eyelid retraction in her right eye. All diagnostic and therapeutic procedures were in accordance with the ethical standards of the institutional and national research committee and with the 1964 Helsinki Declaration and its later amendments or comparable ethical standards. Informed consent was obtained from the participants included in the study.

**Figure 1:**
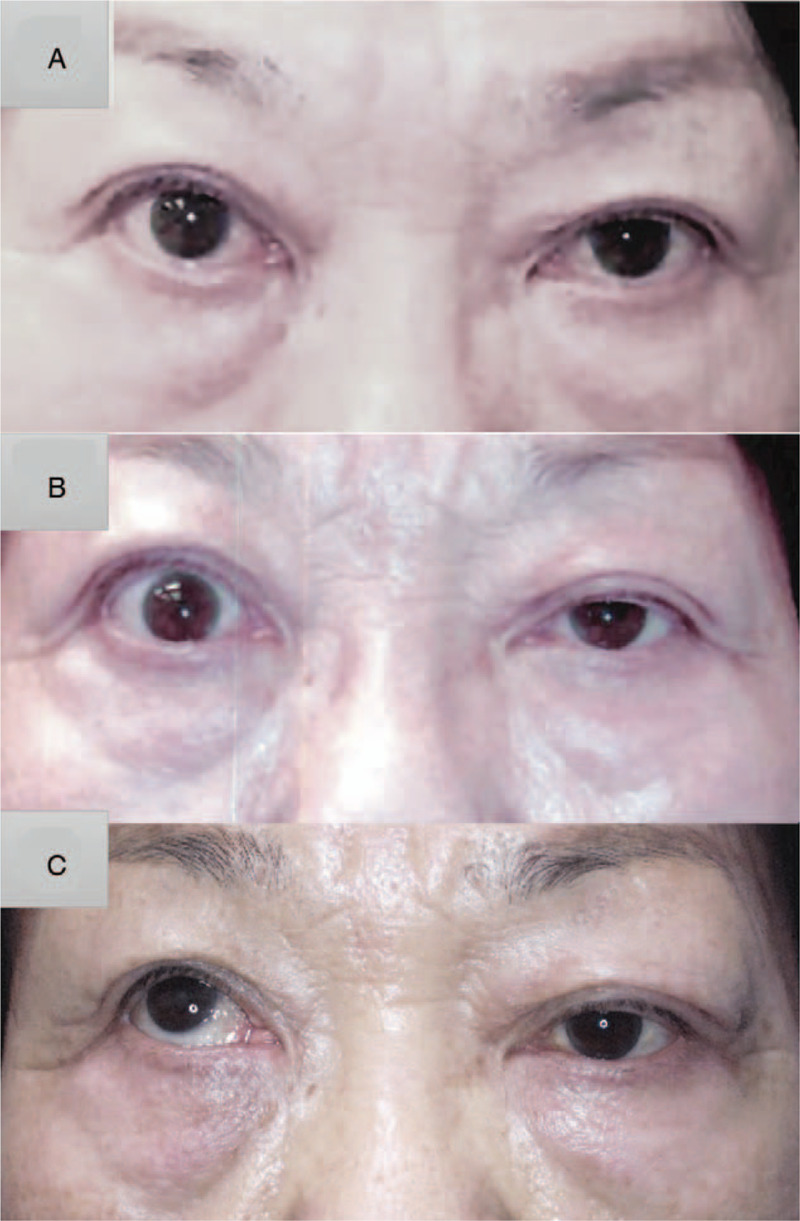
Clinical photographs of the patient. A. Image obtained at initial presentation showing that the right upper eyelid is retracted. The margin reflex distance-1 (MRD-1) of her right eye and left eye was 3.2 mm and 2.1 mm, respectively. B. Image obtained at 3 months post initial presentation showing progression of the right-eye upper retraction. MRD-1 of her right eye was 7.3 mm. C. Image obtained at 6 months post initial presentation showing the development of hyper and exotropia in the right eye.

**Figure 2:**
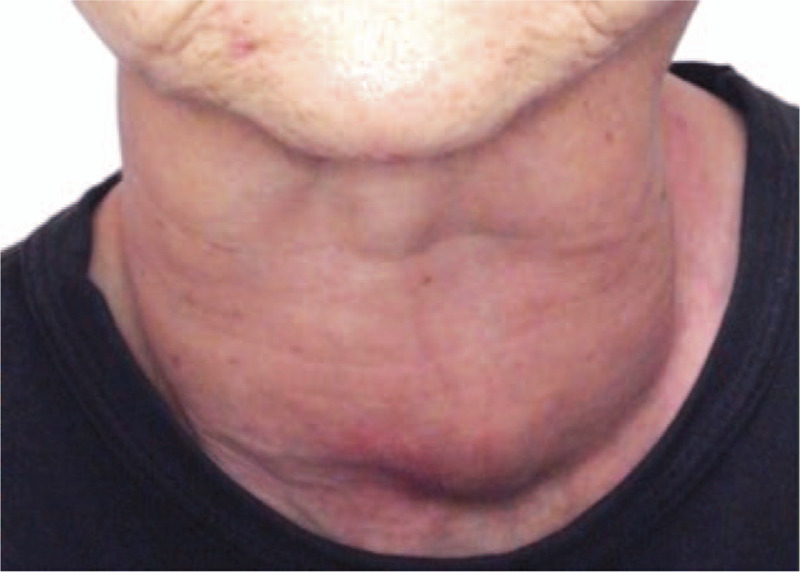
Clinical photographs of the patient showing a prominent enlarged goiter. An ultrasound scan revealed that the goiter was 188.05cc in size, and it was not removed.

**Figure 3:**
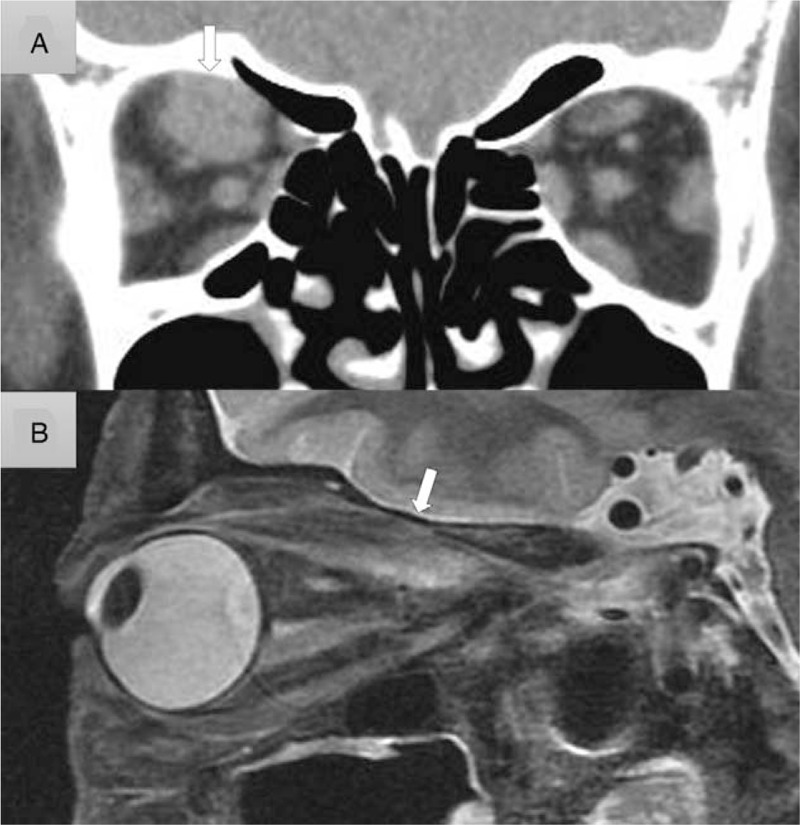
Series of clinical imaging scans showing the development of compressive optic neuropathy (CON). A. Coronal imaging of a computed tomography (CT) scan shows the distinguished right superior levator / superior rectus (SL/SR) muscle complex enlargement (arrow) that caused CON. B. Sagittal imaging of the MRI scan shows that the belly of enlarged SL/SR muscle complex (arrow) is proximate to the orbital apex and is apparently compressing the optic nerve.

Upon initial examination, we found that the margin reflex distance-1 (MRD-1) in her right eye and left eye was 3.2 mm and 2.1 mm, respectively (Fig. 1A). Exophthalmometry findings revealed that the degree of forward displacement was normal (13.5 mm) in her right eye and 12.5 mm in her left eye; no diplopia or visual dysfunction was observed. Her blood test revealed a value of free thyroxine (FT_4_): 1.27 ng/dl (normal range:0.9–1.7 ng/dl), FT_3_: 6.37 pg/ml (normal range: 2.3–4.0 pg/ml), and thyroid-stimulating antibody (TSAb): 1557% (normal range: 0%–120%), and thyroid-stimulating hormone: 0.008 μU/L (normal range: 0.5–5.0 μU/L). The patient stated that she was unable to quit smoking, that is, 1 cigarette per day, and that her husband also smoked, that is, more than 1 pack per day. Her goiter was swollen as much as 188.05cc (Fig. 2), yet she had denied requests to have it removed. At 3 months after her initial presentation, her thyroid hormone control worsened to FT_4_: 3.88 ng/dl, FT_3_: 24.90 pg/ml, TSAb: 3185%, and her upper-eyelid retraction progressed to 7.3 mm of her right-eye MRD-1 (Fig. 1B). After an additional 3 months of insufficient hormonal control, her MRD-1 worsened to 7.7 mm and hyper and exotropia developed, that is, 12 prism diopters (PD) of exotropia and 20PD of right hypertropia during distant gaze (Fig. 1C). As a result, CON developed in her right eye, and the corrected visual acuity in that eye was significantly reduced to 20/200 and the central critical flicker fusion frequency was 16 Hz. Computed tomography (CT) and magnetic resonance imaging (MRI) scans showed a high degree of inflammation and hypertrophy only in the superior rectus and upper levator muscles (Fig. 3).

**Figure 4:**
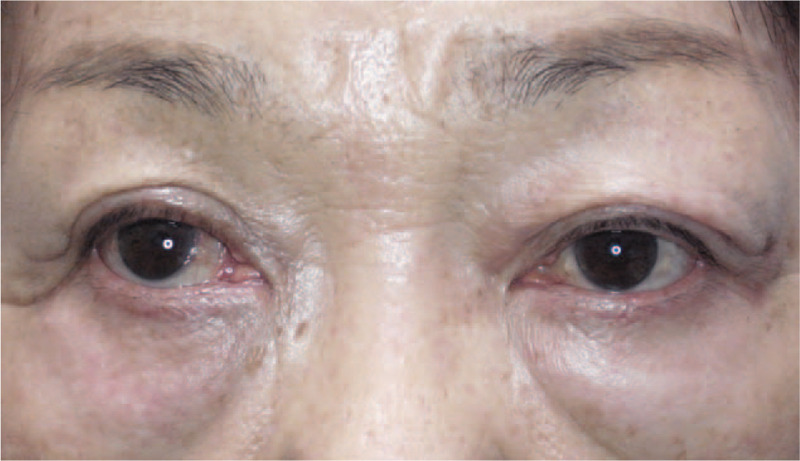
Clinical photographs of the patient obtained at 1-year postoperative. Improvement of the right upper-eyelid retraction and hypertropia can be seen, however, exotropia remained.

Written informed consent was provided by the patient after a comprehensive explanation of the procedures, and intravenous steroid pulse therapy, that is, a daily dose of 500 mg of methylprednisolone every 3 days per week for 2 weeks, and radiation therapy (20GY/10fr) was administered. After medical intervention, the imaging scans revealed no inflammation and her visual acuity recovered to 20/20; however, the right upper-eyelid retraction, that is, MRD-1, 5.9 mm, and hypertropia of 25PD remained. After undergoing the treatment for 9 months, she effectively recovered from her active phase of thyroid ophthalmopathy (FT_4_: 0.5 ng/dl, FT_3_: 2.33 pg/ml, TSAb: 1428%), and we performed bilateral orbital decompression to prevent recurrence of optic neuropathy and to improve any disfigurement. Orbital decompression was performed on the right eye by the deep lateral wall and medial wall, and on the left eye by deep lateral wall resection. As a result, proptosis recovered to 11 mm in the right eye and 12.5 mm in the left eye. At 3-months months after orbital decompression, we performed SL/SR muscle recession, that is, 5 mm and 10 mm, respectively. The muscles were firm and highly fibrotic; however, other peculiar findings were not observed by histopathological investigation, which concurs with that of thyroid orbithopathy. The MRD-1 and hypertropia improved to 3.2 mm from 5.9 mm and to 12PD of exotropia and 10PD of right hypertropia from 10PD of exotropia and 35PD of right hypertropia, respectively (Fig. 4). In all interventions above, no adverse event occurred. The goiter has not yet been removed and the thyroid hormones are regulated around approximately FT_4_ 0.1 ng/dl, FT_3_ 3.0 pg/ml, and TSAb 3000% to 4000%, however, there has been no recurrence of CON for over 1.5 years.

## 4 Discussion

In the current case, the patient had a triad of severe Graves‘ ophthalmopathy, high FT_3_ level, large thyroid, and cigarette smoking,^[2]^ and the FT_3_ level correlated with the onset of worsening ophthalmopathy. The mechanism of extraocular muscle hypertrophy in patients with thyroid-associated ophthalmopathy is the promotion of hyaluronan accumulation between muscle fibers and muscle fibrosis due to activation of orbital fibroblasts via stimulation of anti-thyrotropin-receptor antibodies and an insulin-like growth factor receptor.^[4]^ On the other hand, the mechanism of upper-eyelid retraction is due to levator muscle enlargement similar to the extraocular muscle in over 80% of the cases, and it has been inferred that in the cases without muscle change observed in diagnostic imaging, thyroid hormone and plasma catecholamine stimulate the Mueller's muscles.^[4–9]^ In the present case, the colossal thyroid gland was not removed, immunoreactions were activated, and FT_3_ was supplied to the Mueller's muscles. We theorize that this triggered the progressive upper eyelid retraction that preceded the inflammation and enlargement of the Mueller's muscle, which corresponds to the report of an animal study regarding the relationship between FT_3_ and smooth muscle development.^[10,11]^ Inflammation, which can be particularly severe in tobacco smokers, of the SL can infiltrate the SR through the 2 muscle connections within the same sheath, which explains the time lag between the onset of upper eyelid retraction and hypertropia, and the muscle complex can become enlarged to compress the optic nerve independently.^[1,4]^ However, this fails to explain why the CON in this case was unilateral. Reportedly, unilateral thyroid ophthalmopathy can occur in approximately 10% of cases, with 50% of these cases ultimately shifting to bilateral.^[12–14]^ The transition period may be long and possibly take up to 7 years to occur,^[14]^ thus illustrating that further follow-up examinations should be performed on the fellow eye in the present case. Moreover, the number and sensitivity of the muscles to the hormone and orbital fibroblasts may affect the phenotype. Hence, in our current rare case, we need to follow the transition of thyroid hormones and eyelid changes in detail, in order to address the hypothesis stated above. Moreover, the TSAb interaction has been reported to develop an inflammatory pathway in orbitopathy and to have a correlation with FT_3_, which was also high in the current case.^[4]^ We emphasize that not only TSAb but also FT_3_ levels are essential information for patients to develop severe orbitopathy. Further research on the relationship between thyrotoxicosis and upper eyelid retraction may elucidate more details that could help prevent the onset of the most common symptoms of thyroid ophthalmopathy.

Enlargement of the medial rectus has been focused that it is the most predominant muscle in the factor of CON, however, several recent reports have focused on the enlargement of the levator muscle in thyroid eye disease, and some authors have stated a relationship between the SL/SR muscle complex and CON.^[7,15–17]^ In a volumetric study focusing on the orbital apex, the authors reported that only the specific enlargement of the SL/SR complex makes a significant contribution to CON.^[17]^ In that report, the authors cautioned that the enlargement of the complex substantially contributes to orbital crowding, as the muscle belly of the complex is most proximate to the orbital apex. In the present case, we found that considerable enlargement of the SL/SR complex induced CON, which has not been previously reported, with apparent compression of the optic nerve by the enlarged muscle belly of the complex (Fig. 3). Thus, we suggest that strict attention should be paid to patients with progressive eyelid retraction and hypertropia, and the orbital apex should be evaluated via CT and MRI scanning in order to predict CON, even in cases without proptosis.

Conventional intravenous steroid pulse therapy and radiotherapy were effective in relieving CON, and orbital decompression could prevent recurrence, even in the current characteristic case.^[18]^ The SL/SR complex was the only predominant muscle in the CON group, in which decompression of the inferior wall is considered to be the most effective; however, single wall decompression is likely to recur.^[19]^ In addition, since decompression of the inferior wall tends to cause double vision after surgery,^[20]^ we performed balanced decompression of the lateral and medial wall.^[21,22]^ Regarding upper eyelid retraction and hypertropia, we performed a recession of both muscles simultaneously. This was due to the fact that both muscles are in the same complex that moves cooperatively, unlike other extraocular muscles. Therefore, the overall treatment plan was successful.

In conclusion, in cases of thyroid ophthalmopathy with FT_3_-thyrotoxicosis and giant goiter and with exposure to tobacco smoke, strict attention should be paid to progressive upper eyelid retraction and hypertropia, which may be a significant sign of the development of CON. In addition, regulation of FT_3_ may play an essential role in preventing eyelid retraction and further development of the disease.

## Acknowledgment

The authors wish to thank John Bush for the language editing.

## Author contributions

**Conceptualization:** Masashi Mimura, Hidehiro Oku.

**Data curation:** Masashi Mimura.

**Methodology:** Masashi Mimura.

**Project administration:** Masashi Mimura.

**Supervision:** Hidehiro Oku, Tsunehiko Ikeda.

**Writing – original draft:** Takahisa Hirokawa, Masashi Mimura.

**Writing – review & editing:** Masahiro Tonari, Yohei Sato, Yasushi Fujita, Junko Matsuo, Jun Sugasawa, Tsunehiko Ikeda.
